# Transient Anomalous Diffusion MRI Measurement Discriminates Porous Polymeric Matrices Characterized by Different Sub-Microstructures and Fractal Dimension

**DOI:** 10.3390/gels8020095

**Published:** 2022-02-04

**Authors:** Marco Palombo, Andrea Barbetta, Cesare Cametti, Gabriele Favero, Silvia Capuani

**Affiliations:** 1 Cardiff University Brain Research Imaging Centre, School of Psychology, Cardiff University, Maindy Road, Cardiff CF24 4HQ, UK; palombom@cardiff.ac.uk; 2 School of Computer Science and Informatics, Cardiff University, Maindy Road, Cardiff CF24 4HQ, UK; 3 Chemistry Department, Sapienza University of Rome, Piazzale Aldo Moro 5, 00185 Rome, Italy; andrea.barbetta@uniroma1.it; 4 Physics Department, Sapienza University of Rome, Piazzale Aldo Moro 5, 00185 Rome, Italy; cesare.cametti@uniroma1.it; 5 Department of Environmental Biology, Sapienza University of Rome, Piazzale Aldo Moro 5, 00185 Rome, Italy; gabriele.favero@uniroma1.it; 6 National Research Council—Institute for Complex Systems (CNR-ISC) c/o, Physics Department, Sapienza University of Rome, Piazzale Aldo Moro 5, 00185 Rome, Italy; 7 CREF, Museo Storico Della Fisica e Centro Studi e Ricerche Enrico Fermi, 00185 Rome, Italy

**Keywords:** porous polymeric matrices, fractal dimension, diffusion NMR, anomalous diffusion, sub-diffusion, porosity, dielectric spectroscopy

## Abstract

Considering the current development of new nanostructured and complex materials and gels, it is critical to develop a sub-micro-scale sensitivity tool to quantify experimentally new parameters describing sub-microstructured porous systems. Diffusion NMR, based on the measurement of endogenous water’s diffusion displacement, offers unique information on the structural features of materials and tissues. In this paper, we applied anomalous diffusion NMR protocols to quantify the subdiffusion of water and to measure, in an alternative, non-destructive and non-invasive modality, the fractal dimension d_w_ of systems characterized by micro and sub-micro geometrical structures. To this end, three highly heterogeneous porous-polymeric matrices were studied. All the three matrices composed of glycidylmethacrylate-divynilbenzene porous monoliths obtained through the High Internal Phase Emulsion technique were characterized by pores of approximately spherical symmetry, with diameters in the range of 2–10 μm. Pores were interconnected by a plurality of window holes present on pore walls, which were characterized by size coverings in the range of 0.5–2 μm. The walls were characterized by a different degree of surface roughness. Moreover, complementary techniques, namely Field Emission Scanning Electron Microscopy (FE-SEM) and dielectric spectroscopy, were used to corroborate the NMR results. The experimental results showed that the anomalous diffusion α parameter that quantifies subdiffusion and d_w_ = 2/*α* changed in parallel to the specific surface area S (or the surface roughness) of the porous matrices, showing a submicroscopic sensitivity. The results reported here suggest that the anomalous diffusion NMR method tested may be a valid experimental tool to corroborate theoretical and simulation results developed and performed for describing highly heterogeneous and complex systems. On the other hand, non-invasive and non-destructive anomalous subdiffusion NMR may be a useful tool to study the characteristic features of new highly heterogeneous nanostructured and complex functional materials and gels useful in cultural heritage applications, as well as scaffolds useful in tissue engineering.

## 1. Introduction

The measurement of molecular diffusion by nuclear magnetic resonance (NMR) techniques is an effective experimental method to probe biological and porous material structures, since the meso- and microstructural features of highly heterogeneous systems can be characterized by diffusing small molecules (typically water) within them. 

Nowadays, the measurement of water molecules in highly heterogeneous and complex systems using diffusion-weighted NMR (DW-NMR), also in the imaging modality (magnetic resonance imaging, MRI), has broad applications developed on the basis of seminal works in material science [1,2,3,4,5], biophysics [6,7,8] and medicine, especially in the imaging modality [8,9,10]. The non-invasive observation of endogenous water’s diffusion displacement provides a source of contrast for MRI, which offers unique information on the subtle structural features and topological organization of materials and tissues [11,12]. 

In soft condensed matter, the length scale (l_D_) by which we can probe media structure is approximately equal to the square root of the mean-square displacement (MSD) of the diffusing particles: l_D_ ≈ MSD^1/2^. In the normal Brownian diffusion approximation, the MSD of diffusing water molecules is linearly proportional to the diffusion coefficient D and the time t during which the diffusion process is observed (MSD ∝ D · t) [13]. Taking typical values for free water diffusion and diffusion time achievable on NMR scanners (e.g., D ≈ 3 μm^2^/ms and t ≈ 10–100 ms), molecular displacements occur over linear distances of about 10–40 micrometers. This distance, which represents the intrinsic resolution of conventional Brownian DW-NMR investigation, is orders of magnitude smaller than the macroscopic MRI resolution (usually 1–2 mm in clinical applications and about 100 micrometers in microimaging research investigations).

However, considering the current development of new nanostructured and complex materials, it is critical to develop a sub-micro-scale sensitivity to quantify experimentally new parameters characterizing sub-microstructured porous systems. In this regard, NMR diffusion techniques that use high magnetic field gradient strength have been developed to both increase the image resolution (i.e., decrease the voxel size) [14] and to exploit the phenomenon of diffusion-diffraction [2,15,16,17,18,19,20] to obtain sub-microstructural information. However, these methods can only be used in materials science or in ex vivo experiments. In fact, there is a limit to the maximum strength of magnetic field gradients that can be used in the clinic application. This limit implies the impossibility of using the aforementioned methods [2,14,15,16,17,18,19,20] and the impossibility of obtaining clinical images with a voxel size lower than 0.3 × 0.3 × 0.3 mm^3^. In the latter case, the intrinsic resolution provided by l_D_ can be exploited to obtain submicroscopic information. 

Nowadays, in the innovative field of tissue engineering, different kinds of scaffolds, i.e., temporary support that aids seeded cells to proliferate and reorganize themselves into tissue-like structures, hold a strategic position [21]. Several different polymeric scaffolds have been developed with the final goal of producing better nano- and micro-architectural features to promote cell interaction and tissues formation [22,23]. On the other hand, the development of advanced materials and methodologies for the cleaning and protection of works of art, such as gels [24], nanostructured cleaning fluids [25], composites and other functional materials [26], represents the major trends in conservation science.

It is therefore highly desirable to develop an experimental non-invasive and non-destructive method for the structural characterization of porous polymeric matrices in their swollen state that is capable of probing them over a wide range of length scales (from 10 nm to 100 μm). 

An effective approach to obtain geometrical and topological information from porous heterogeneous media, based on transient anomalous diffusion (AD) by NMR [27,28,29,30], was introduced.

Nowadays, it is recognized that anomalous subdiffusion [13] exists in complex and highly heterogeneous media, such as biological tissues [31] and biomaterials [32,33]. Therefore, anomalous subdiffusion is a probe of submicroscopic organization, as shown in theoretical [34] and experimental works performed by optical microscopy [35,36,37,38]. Specifically, anomalous subdiffusion is characterized by an MSD of diffusing particles growing nonlinearly in time, i.e., MSD ∝ t^α^ (with α < 1) [13]. The α parameter, which quantifies subdiffusion processes due to entrapped diffusion in multicomponent or hierarchical structures and boundary micro-roughness, is sensitive to sub-microstructures occurring at different length scales. Moreover, in specific cases, the α measurement is related to fractal dimension d_w_ [39]. Importantly, the l_D_ sizes associated with subdiffusion processes are smaller than those of Brownian DW-NMR investigations.

In this paper, we proposed and tested the potential of anomalous subdiffusion NMR imaging measurement (α-imaging) to investigate porous polymeric matrices characterized by different sub-microstructures.

To this end, we investigated three different polymeric matrices presenting different submicroscopic morphologies. Specifically, three chemically homogeneous specimens, constituted of the same voids of about 10 μm in diameter coexisting with different smaller pores (few microns and hundred nanometers in size) present within the walls, were characterized, including the evaluation of their fractal dimension, by techniques complementary to NMR, namely Field Emission Scanning Electron Microscopy (FE-SEM) and dielectric spectroscopy. Then, conventional Brownian-based diffusion MRI and anomalous subdiffusion (α-imaging) were performed. Our results showed that α-maps discriminated between polymeric matrices based on their sub-microstructural characteristics. In contrast, conventional DW-NMR was not able to do so. Therefore, anomalous subdiffusion NMR imaging measurement, being a non-invasive and radiation-free approach, may be a useful technique to select the activity and monitor in vivo the choice of polymeric scaffolds in different biotechnological applications, such as tissue engineering. On the other hand, the non-invasive and non-destructive anomalous subdiffusion NMR may be a useful tool to study the characteristic features of new highly heterogeneous nanostructured and complex functional materials.

## 2. Results and Discussion

### 2.1. FE-SEM and Dielectric Spectroscopy Results 

**Figure 1:**
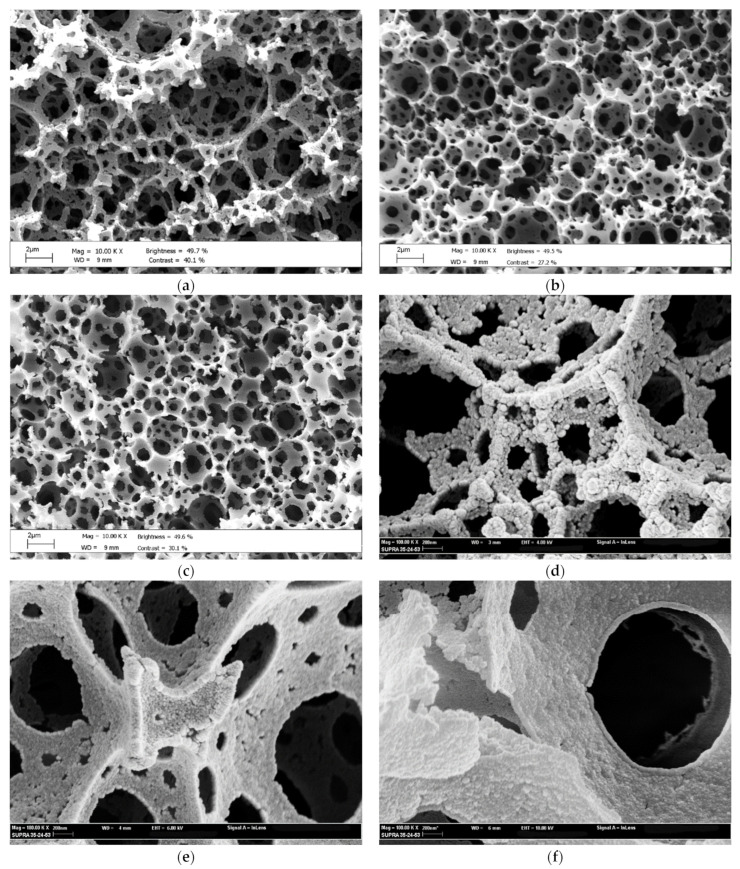
Scanning Electron (SEM) (**a**–**c**) and Field Emission Scanning Electron (FE-SEM) (**d**–**f**) micrographs of glycidylmethacrylate-divynilbenzene porous monoliths obtained through the High Internal Phase Emulsion (HIPE) technique. Sample in (**a**,**d**): GMA/DVB 80:20 (G80); sample in (**b**,**e**): GMA/DVB 60:40 (G60); sample in (**c**,**f**): GMA/DVB 40:60 (G40). Scale bars: (**a**–**c**), 2 μm; (**d**–**f**), 200 nm. As evidenced from the high magnification micrographs (**d**–**f**), the samples have a granular texture. The dimension of particles decreases with the increase of the content of crosslinker.

PolyHIPEs are obtained by polymerizing the external phase of a high internal phase emulsion, i.e., an emulsion in which the volume fraction of the internal phase (Φ) exceeds the critical value of 0.74. Above this value, the dispersed phase droplets are tightly packed and adopt a polyhedric shape. Crosslinking of the continuous phase causes a contraction in the volume of the polymer network and the appearance of interconnecting holes at the contact areas where droplets are separated by a film of continuous phase a few nm in thickness. The SEM micrographs in Figure 1a–c illustrate the typical morphology of polyHIPEs represented by approximately spherical voids a few μm in size interconnected by a plurality of window holes. The decrease in void diameter as the content of GMA decreases is worth noting (Figure 1a–c, GMA/DVB 80:20 > GMA/DVB 60:40 > GMA/DVB 40:60). An additional porosity level in the meso- and micro-scale length can be introduced within the polyHIPE walls by blending the monomer phase (GMA + DVB)) with a thermodynamically compatible, inert solvent that acts as a porogen (toluene). During the first stage of polymerization, a high number of nuclei form which agglomerate in a later stage of the monomer conversion, forming a continuous polymeric network. Pores are located within and in between agglomerates. The higher the crosslinker content, the earlier the phase separation occurs when nuclei are still very small. Consequently, pores within and in between agglomerates decrease in size as the crosslinker concentration increases [40]. The microstructure of the polyHIPEs, shown in Figure 1d–f, is in accordance with such a mechanism. PolyHIPEs with a lower percentage of DVB (GMA/DVB 80:20) present a surface texture made of relatively large particles, and large pores are clearly discernible. As the content of DVB increases, the particles size decreases, walls appearance becomes denser and pores become less and less apparent (e.g., GMA/DVB 40:60). The surface areas reported in Table 1 are coherent with such a morphological analysis.

**Figure 2:**
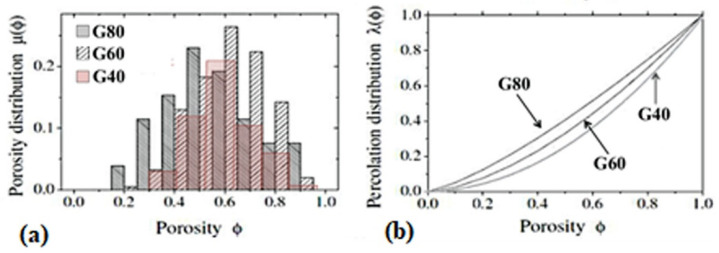
The local porosity distribution (**a**) and the local percolation probability (**b**) as a function of the porosity for samples GMA/DVB 80:20 (G80), GMA/DVB 60:40 (G60) and GMA/DVB 40:60 (G40), deduced by dielectric relaxation measurement.

Specifically, as reported in the Materials and Methods section, the samples were identified by different specific surface area S from 16 m^2^/g to 188 m^2^/g, reflecting different local percolation probabilities λ(ϕ) and local porosity distributions μ(ϕ). S, μ(ϕ) and λ(ϕ) are used to characterize the connectivity in porous and highly heterogeneous media together with transport properties prediction [41]. Figure 2 shows the local porosity distribution μ(ϕ) and the local percolation probability λ(ϕ) of the three polymeric matrices investigated by dielectric relaxation spectroscopy measurement [42]. Among the investigated samples, the G40 sample is characterized by the largest S and the lowest percolation probability.

All the three matrices are characterized by pores of approximately spherical symmetry of the diameter in the range of 2–10 μm (Figure 1a–c). Pores are interconnected by a plurality of window holes present on pore walls, which are characterized by size coverings in the range of 0.5–2 μm. A close inspection of the wall morphology (Figure 1d–f) reveals a different degree of surface roughness. The surface topography is a manifestation of the inner structure of the materials. The presence of cross-linkers in the HIPE formulation is responsible for phase separation, at a certain degree of monomer-cross-linker conversion, of the polymeric network into the form of microgels swollen with porogen and left monomer phase. The microgels assembly is then bound together to form the observed structure. The higher is the starting cross-linker content, the earlier is the onset of phase separation, and the more numerous and smaller are the dimensions of the microgels [40,43]. This is reflected in physical characteristics of the final material, such as surface area S and surface fractal dimension d_w_. It is intuitive that the smaller the size of the particles composing the material, the smaller the fractal dimension of the walls (or the less the degree of roughness of surface walls). The data reported in Table 1 of the Material and Methods section provide quantitative evidence of the pictures displayed in Figure 1. The surface areas of the matrices are directly proportional to the divynilbenzene (DVB) content, while the average pore diameter of pores below 0.2 μm follows an inverse relationship.

### 2.2. Conventional Normal and Anomalous Diffusion MRI 

**Figure 3:**
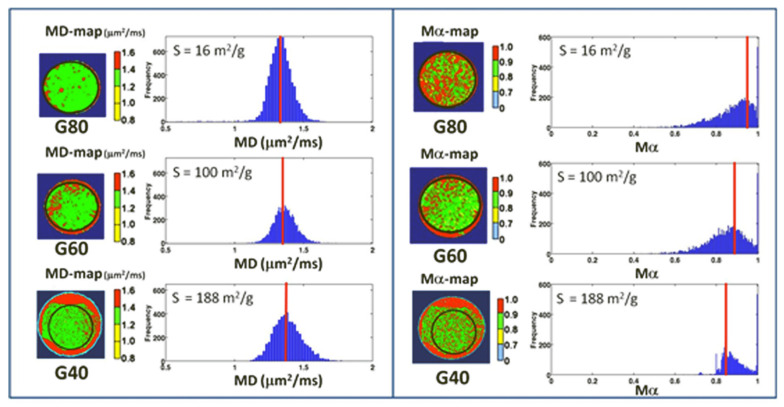
(**Left**): Conventional diffusion MRI based on Brownian diffusion. MD-maps obtained at diffusion time t = Δ = 100 ms, for the three polymeric matrices samples (G80, G60, G40), together with the measured distribution of MD values in the ROI bordered by the black circles, are reported. Red straight lines indicate the median = mean value. (**Right**): Anomalous diffusion MRI. Mα-maps obtained for G80, G60 and G40 samples, together with the measured distribution of Mα values in the ROI bordered by the black circles. Red straight lines indicate the median value. The results displayed here suggest that MD is not sensible to sub-microstructure variations. Conversely, Mα discriminates among polymeric matrices characterized by different specific surface areas *S* (m^2^/g).

Conventional mean diffusivity (MD) maps [9,12] based on Brownian diffusion and mean α (*M*α) maps based on anomalous subdiffusion were obtained for all the three G80, G60 and G40 samples. Figure 3 shows the color-coded maps related to conventional DW MRI (left panel) and α-imaging (right panel), together with the measured distribution of MD and Mα values extracted from the region of interest (ROI) bordered by the black circles. The median value of MD (represented by the red line in Figure 3, left panel) does not significantly change with the specific surface area *S* of the investigated polymeric matrices. Conversely, as shown in the right panel of Figure 3, the *M*α values change in parallel to the *S* of the porous matrices. In particular, the *M*α values are distributed around different values. *M*α values = 1 suggest that the majority of water diffuses within the large pores and interconnections with little to negligible interaction with the fractal surface. In contrast, *M*α values < 1 suggest that a significant proportion of water molecules diffuse in the proximity of and within the microstructures comprising the roughness of the pore walls. The lower the *M*α values, the higher the proportion of water molecules whose diffusion is impacted by the fractal surface.

### 2.3. Quantitative and Comparative Analysis 

**Figure 4:**
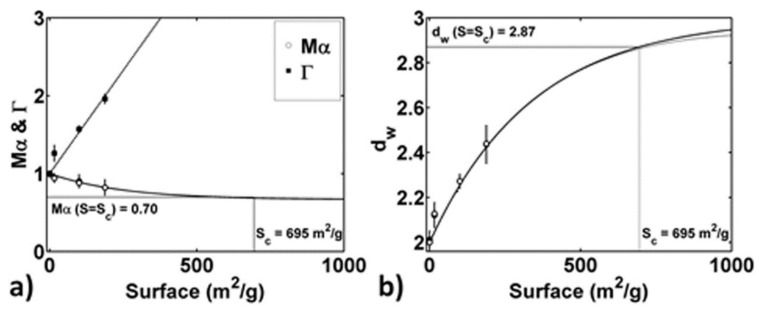
(**a**) mean values of *M*α distribution belonging to the diffusing water pool interacting with pore walls and the values of Γ scaling exponent estimated from dielectric spectroscopy measurements are reported as a function of the pores surface area, *S*. Dashed line represents linear trend of data, while straight line represents the exponential decay function: *M*α = (1 − *Mα_c_*)exp[−*S*/*σ*] + *Mα_c_* fitted to the data. The experimental point at *S* = 0 and Mα = 1 is related to Brownian diffusion of free water. The limiting value of *S* at the percolation threshold is extrapolated from the fit at Mα = Mαc = 0.7. (**b**) d_w_ values corresponding to Mα values are reported as a function of *S*. Straight line represents the function: d_w_ = 2/*M*α = 2/{(1 − *Mα_c_*)exp[−*S*/*σ*] + *Mα_c_*}, while dashed line represents its Pade approximant: d_w_ = (2 + *2x* + *x*^2^/6)/(1 + *x*/2 + *x*^2^/12), with *x* = *S*/*σ*. The experimental point at *S* = 0 and d_w_ = 2 is related to Brownian diffusion of free water. The limiting value of *S* at the percolation threshold is extrapolated from the fit at d_w_ = 2.87.

We focused our quantitative analysis of anomalous subdiffusion related to pore surface fractal dimension according to the equivalence: d_w_ = 2/*M*α. The mean values of *M*α distribution for the three samples are reported in Figure 4a and compared with the power-law exponent Γ (linking the percolation probability λ to the surface porosity ϕ) as measured by dielectric spectroscopy. In Figure 4b, relative derived d_w_ values are reported as a function of S.

In the investigated samples, the system is far from the value of *S*, determining a percolation cluster at the critical percolation threshold, S_c_. Indeed, the limiting value for the fractal exponent d_w_, and thus for α, are well known from the percolation theory. At the percolation threshold S_c_, d_w_ = 2.87 and α_c_ ≈ 0.70 for particles diffusing on the infinite cluster [44], while at *S* = 0 (i.e., the surface area of smooth pores), diffusion is normal and d_w_ = 2, i.e., α = 1. Near the percolation threshold, some diffusing particles are trapped in bounded regions. Well above the percolation threshold, all the diffusing particles are trapped. From the α and d_w_ values reported in Figure 4a,b, it is clear that all the samples investigated are below the percolation threshold value of *S*, because d_w_ < 2.87 and *Mα >* 0.70. It is possible to estimate the limiting value of *S* for a porous matrix characterized by the same dimension of pores and interconnections, as well as the same density, but differently smooth walls, using the empirical relation:

(1) $$M\alpha =\left(1-M\alpha_{\infty }\right)\exp\left(-\frac{S}{\sigma }\right)+M\alpha_{\infty }$$

fitted to the data in Figure 4a with *M*α_∞_ ≈ 2/3. The result of the fitting procedure suggests that *S*_c_ = *S* (*M*α = 0.7) ≈ 695 m^2^/g. Moreover, from Figure 4b, it is possible to highlight that relation (1) can be successfully used to describe the behavior of d_w_ = 2/*M*α as a function of *S*.

The results obtained here are fully consistent with those obtained by Saxton, who used numerical simulations of particle diffusion in percolating clusters [45]. Indeed, Saxton’s results [45] show an equivalent trend for d_w_ as a function of obstacle concentration *C* for different kinds of obstacle spatial distributions. Saxton described the relation between d_w_ and *C* as:

(2) $$d_{w}=\frac{2+A_{1}x+B_{1}x^{2}}{1+A_{2}x+B_{2}x^{2}}$$

with *x* = *C*/*C*_0_. In this work, the role of obstacle concentration was played by the pore’s surface area *S*, and the relation (2) between d_w_ and *S* can be obtained by the Pade approximant of relation (1), with *x* = *S*/*σ* (dashed line in Figure 4b). The good agreement between relations (1) and (2) and the experimental data (Figure 4b) confirms the correctness of fractal dimension estimation obtained from the anomalous subdiffusion of water quantified by MRI. 

### 2.4. Further Considerations and Perspectives

Moving away from the classical theory of diffusion in fractals and disordered media [46], a promising avenue to pursue in future work is the use of the Feynman-Enderlein path integral as a powerful method to model the complex dynamics of water molecular diffusion in highly heterogeneous and complex multiscale media such as those investigated here. However, the complete characterization of water pools with all possible paths is a formidable challenge, mostly due to track ergodicity, especially in fractal systems. Typically, only a very limited number of paths can be measured directly through single-particle tracking techniques. Alternatively, only a very coarse statistical description of the average dynamic can be indirectly obtained through techniques such the presented DW-NMR ones. This challenge could be addressed using recent techniques based on a deep-learning Feynman’s formulation with a pre-classification scheme that can directly predict the final results only with (a few) data of initial conditions [47].

On the other hand, the polymeric matrices used and described here could be of interest for the development of new micro-nanostructured dielectric metasurfaces [48].

## 3. Conclusions

In conclusion, we showed that the DW-NMR imaging procedure for quantifying the subdiffusion of water (or liquids) in highly heterogeneous porous systems can be used to measure, in an alternative, non-destructive and non-invasive modality, the fractal dimension of systems characterized by micro and submicro geometrical structures. The experimental results obtained in porous polymeric matrices characterized by different sub-microstructures and fractal dimensions suggest that the NMR method tested may be a valid experimental tool to corroborate different theories and simulations developed and performed for describing highly heterogeneous and complex systems. On the other hand, non-invasive and non-destructive anomalous subdiffusion NMR may be a useful tool to study the characteristic features of new highly heterogeneous nanostructured and complex functional materials and gels useful in cultural heritage applications. Moreover, anomalous subdiffusion MRI measurement, being a radiation-free technique, may have a wide range of applicability and potential to monitor in vivo polymeric scaffolds in different biotechnological applications, such as tissue engineering.

## 4. Materials and Methods

### 4.1. Theoretical Background 

#### 4.1.1. Characterization of Heterogeneous Systems by Dielectric Relaxation Spectroscopy Measurement

In the framework of the effective medium approximation theory of heterogeneous media, such those we are dealing with, the geometrical characterization of the system, from a pure phenomenological point of view, occurs through a single parameter, the porosity Φ, defined as the total volume of the pore space divided by the total volume of the sample under test. Clearly, as described by Hilfer [49], this parameter alone cannot suffice to characterize the pore space geometry.

Hilfer [41], on the basis of the local porosity theory, described the porosity of a heterogeneous mixture through two functions, i.e., the local porosity distribution μ(ϕ) and the local percolation probability λ(ϕ). The first function gives the probability density of finding a porosity ϕ between ϕ and ϕ+dϕ inside the sample and is related to the bulk average porosity Φ (the volume fraction of water in the whole sample in our case) through the expectation value

(3) $$\Phi =\int_{0}^{1}\phi \mu (\phi )d\phi$$

The second function λ(ϕ) denotes whether the pore space percolates or not, i.e., this parameter gives the fraction of percolating cells with a local porosity ϕ. This function characterizes the property of whether a path exists connecting a point to any of the others, completely inside the pore phase.

These two functions can be experimentally resolved by analyzing the dielectric response of the heterogeneous system under a wide enough frequency range, where the frequency-dependent complex dielectric constant ε*(ω) experiences the interfacial polarization dispersion.

Following the procedure proposed by Hilfer [41], we measured the dielectric properties of different polymeric structures and characterized by a different average porosity Φ and different connectedness of the porous medium. 

The analysis of the dielectric response, on the basis of Equation (3), allows us to obtain both the functions μ(ϕ) and λ(ϕ).

In the light of the mixing law of local porosity theory [41,49], the complex dielectric constant ε*(ω) of the whole heterogeneous system, composed by a water phase embedded into a more or less interconnected polymeric matrix, can be obtained from the solution of the integral equation [33]:

(4) $$\int_{0}^{1}\frac{\varepsilon_{we}^{*}(\omega )-\varepsilon (\omega )}{\varepsilon_{we}^{*}(\omega )+2\varepsilon (\omega )}\lambda (\phi )\mu (\phi )d\phi +\int_{0}^{1}\frac{\varepsilon_{pe}^{*}(\omega )-\varepsilon (\omega )}{\varepsilon_{pe}^{*}(\omega )+2\varepsilon (\omega )}(1-\lambda (\phi ))\mu (\phi )d\phi =0$$

where $\varepsilon_{we}^{*}(\omega )$ and $\varepsilon_{pe}^{*}(\omega )$ are the effective complex dielectric constants of the aqueous (conducting) phase and the polymeric (non-conducting) network with a local porosity ϕ.

Both the dielectric constants $\varepsilon_{we}^{*}(\omega )$ and $\varepsilon_{pe}^{*}(\omega )$ depend on the bulk complex dielectric constants of the two components, according to the relationships

(5) $$\begin{array}{l} \varepsilon_{we}^{*}(\omega )=\varepsilon_{w}^{*}(\omega )\left[1-\frac{1-\phi }{{\left[1-\frac{\varepsilon_{p}^{*}(\omega )}{\varepsilon_{w}^{*}(\omega )}\right]}^{-1}-\frac{1}{3}\phi }\right]\\ \varepsilon_{pe}^{*}(\omega )=\varepsilon_{p}^{*}(\omega )\left[1-\frac{\phi }{{\left[1-\frac{\varepsilon_{w}^{*}(\omega )}{\varepsilon_{p}^{*}(\omega )}\right]}^{-1}-\frac{1}{3}(1-\phi )}\right] \end{array}$$

Here, $\varepsilon_{p}^{*}(\omega )$ and $\varepsilon_{w}^{*}(\omega )$ are the complex dielectric constants of the polymeric matrix and of the aqueous phase, respectively. These complex quantities are defined as

(6) $$\varepsilon_{j}^{*}\left(\omega \right)=\varepsilon_{j}(\omega )+\frac{\sigma_{j}}{i\varepsilon_{0}\omega }\;(j=p,w)$$

where the dielectric losses are originated by contributions from the electrical conductivity σ. Because of the range of frequencies investigated here and considering that the porous matrix has negligible electrical conductivity ($\sigma_{p}≃0$) and its permittivity $\varepsilon_{p}$ is practically independent of frequency and temperature, the following approximations hold:

(7) $$\begin{array}{l} \varepsilon_{p}^{*}\left(\omega \right)=\varepsilon_{p}\\ \varepsilon_{w}^{*}(\omega )=\varepsilon_{w}+\frac{\sigma_{w}}{i\varepsilon_{0}\omega } \end{array}$$

When the functions μ(ϕ) and λ(ϕ) are known, the dielectric response of the water embedded polymeric system is described by Equation (4), which furnishes, once numerically solved, the value of $\varepsilon^{*}\left(\omega \right)$ (the real part, the permittivity $\varepsilon^{\prime }\left(\omega \right)$, and the imaginary part, that is, the dielectric loss $\varepsilon^{\prime \prime }\left(\omega \right)$). In our case, however, we must solve the inverse problem, i.e., calculate the two functions $\mu \left(\phi \right)$ and $\lambda \left(\phi \right)$ from the fitting of Equation (1) to the experimental data.

In order to make this task easier, we assumed a well-defined functional form for $\lambda \left(\phi \right)$ and $\mu \left(\phi \right)$. For the percolation probability $\lambda \left(\phi \right)$, we used the power-law function

λ(ϕ)=ϕ^Г^ (8)

With Г as a fitting parameter. As far as the local porosity distribution $\mu \left(\phi \right)$ is concerned, without the lack of generality, we assumed a normal (Brownian) distribution, characterized by a mean value $\phi_{0}$ and by a variance $Var_{\phi }$. In this way, the number of free parameters in the fitting procedure is confined to three, i.e., the exponent Г, the mean value $\phi_{0}$ and the variance $Var_{\phi }$.

#### 4.1.2. Characterization of Heterogeneous Systems by Diffusion-Weighted MRI Measurements: Conventional Normal and Anomalous Diffusion

In this paper, to quantify anomalous subdiffusion and fractal dimension by NMR, we used the method introduced by Palombo and Capuani et al. [27,28,29,30] with minor extensions. The procedure is based on the theory of diffusion in fractals and disordered media, a comprehensive review of which can be found elsewhere [46,49,50,51]. However, a summary of the relevant theory is included in this section for completeness.

In disordered and fractals media, the temporal dependence of the diffusional mean-squared displacement ($MSD=\langle r^{2}\rangle$) is known to deviate from linearity [51], showing a power law in deterministic fractals [52] which accurately describes the temporal scaling behavior of diffusion in realizable environments over a considerable range of length and time scales. For example, in biological media, Saxton [53] showed that the presence of a hierarchy of binding sites leads to a wide anomalous diffusion regime. Generally speaking, this power-law relation can be expressed through the relationship

(9) $$MSD=\langle r^{2}\rangle \propto t^{2/d_{w}}$$

where *t* is the diffusion time, and d_w_ is the fractal dimension of the diffusion process, which can be considered a “statistical fractal.” This point follows from the self-similarity of the random walks: A discrete step taken at one timepoint can be envisioned to be the sum of net displacements taken during smaller time intervals. The above scaling relationship provides a means to categorize different diffusion processes. For Normal (i.e., Brownian) diffusion, d_w_ is exactly 2, which leads to the linear dependence of *MSD* on diffusion time. When the mean-squared displacements increase more rapidly, i.e., d_w_ < 2, the process is in the superdiffusive regime, the opposite case of d_w_ > 2 describes a subdiffusive process.

In general, natural systems can show a range of heterogeneity length scales: l_D_ [44,46,50] between a minimum l_min_, and a maximum l_max_. Both theoretical and experimental studies [30,45,46,53,54,55,56] have shown that three distinct diffusion regimes of solute particles can be identified in highly heterogeneous media. For l < l_min_, diffusion is not sensible to heterogeneities. The ordinary linear relation between $\langle r^{2}\rangle$ and the diffusion time holds with a constant diffusion coefficient D, equal to the bulk diffusivity D_0_, of the medium. On the other hand, when l >> l_max_, caging and trapping effects due to heterogeneities are massively averaged out. The linear relation holds again but with an effective constant diffusion coefficient D_∞_ < D_0_, which considers the average effect of traps and barriers [57]. Finally, on intermediate scales l_min_ ≤ l ≤ l_max_, the diffusion is sensible to heterogeneity variations and may show a more complex behavior, known as transient non-Fickian or anomalous diffusion. A fractional exponent α = 2/d_w_ is often introduced to investigate and classify anomalous diffusion processes. Ordinary (Brownian) diffusion is recovered for α = 1, while subdiffusion processes are characterized by α values lower than 1. Strictly speaking, the term anomalous diffusion refers to an asymptotically (in time and scale) anomalous regime, i.e., the t^α^ behavior should persist for t→∞. This behavior can result from an infinite hierarchy of heterogeneity length scales. However, many real highly heterogeneous and/or complex porous materials, such as those investigated in this study, exhibit a finite hierarchy of heterogeneity length scales, which is a sufficient condition for anomalous diffusion at short times crossing over to normal diffusion at long times. Therefore, in this work, instead of defining away the anomalous diffusion, we analyzed and quantified the initial period of transient anomalous diffusion.

The anomalous diffusion in these kinds of systems can be analyzed using percolation theory [39,44,46,58], where the role of the obstacle concentration *C* is played by the pores surface area *S*. In classical percolation theory, at obstacle concentrations below the critical percolation threshold *C < C_c_*, there is a percolation cluster, that is, a cluster of unobstructed lattice sites (the pores of a porous matrix) that provides a continuous path for long-range diffusion. The percolation cluster is fractal over short distances (l_min_ ≤ l ≤ l_max_), and homogeneous over long distances (l >> l_max_) [58]. The crossover length diverges as the obstacle concentration approaches the percolation threshold *C* *≈ C_c_*.

(10) $$l_{\max}\sim {\left|C-C_{c}\right|}^{-\upsilon +\beta /2}$$

where *ν* = 4/3 and *β* = 5/36 are two-dimensional scaling exponents [45]. The exponent *β* gives the probability that a lattice site is part of the infinite cluster, *P*(∞) ~ |*C* – *C_c_*|^β^. At the percolation threshold, the percolating cluster is self-similar over all length scales, with no characteristic length scale. Because the percolating cluster is self-similar at *C_c_*, the diffusion coefficient at *C_c_* is time-dependent for all times. As time increases, the diffusing particle encounters dead ends, bottlenecks, and other hindrances at longer and longer length scales. A particle may escape a small dead end only to find that it is still trapped in a larger dead end.

The effective fractional exponent α can be measured using pulsed field gradient (PFG) NMR techniques [11,27] by collecting the NMR PFG signal attenuation *S*(*q*, Δ) as a function of the diffusion time Δ, and using the asymptotic expression of the Fourier transform of the motion propagator for the sub-diffusive regime derived in [27]:

(11) $$S\left(q,∆\right)/S\left(q=0,∆\right)\sim \exp\left(-K_{\alpha }q^{2}{∆}^{\alpha }\right)$$

where the wavevector *q* = qn is defined by *q* = 1/2π γgδn, where γ is the gyromagnetic ratio of the water proton, g is the pulsed magnetic field gradient strength, δ is the pulsed magnetic field gradient duration and *n* indicates a specific magnetic gradient direction [11]. The above relation holds when $q^{2}\ll \frac{1}{K_{\alpha }{∆}^{\alpha }}$ is kept constant at varying Δ, with *K_α_* a generalized diffusion constant, and Δ >> δ. The mean α value, Mα, is calculated by averaging α values obtained along the x, y and z directions.

On the other hand, the mean diffusivity (MD) is obtained by diffusion tensor imaging (DTI) [9] protocol that foresees acquisitions along six non-coplanar directions of the magnetic diffusion gradient plus an acquisition in the absence of the diffusion gradient to obtain all the independent elements of the diffusion tensor. MD is the trace of the diffusion tensor whose elements are quantified in each DW image voxel through a fit of a decreasing monoexponential function in diffusion coefficient D and b-value to the diffusion-weighted data. The b-value includes all the PFG parameters selected by the experimenter: b =$\;\left(\gamma g\delta \right)$^2^Δ, where Δ is the diffusion time (assuming that Δ >> δ).

### 4.2. Experimental Section

#### 4.2.1. Materials

Technical-grade divinylbenzene (weight formula = 130.19, Aldrich; 80 vol% m- (55 vol%) and p-divinylbenzene (25 vol%), the remainder being m- and p-ethylstyrene) and glycidyl methacrylate, 97% (Aldrich, weight formula = 142.15) were purified by passing through a column of basic alumina (Brockmann I) to remove the inhibitor (p-tertbutylcatechol in the case of DVB and ether hydroquinone in the case of GMA). Toluene was purchased from Sigma Aldrich and the surfactant PGE 080/D from Danisco, Denmark.

#### 4.2.2. Porous Polymeric Matrices

Three highly porous polymeric matrices with randomly oriented interconnected pores were obtained by high internal phase emulsion templating (HIPE) [59,60]. An aqueous solution of CaCl_2_ (1.1 wt%) and potassium persulfate (0.22 wt%) was dispersed in the form of discrete droplets in an organic phase composed by glycidylmethacrylate (GMA), divinylbenzene (DVB), a porogen (toluene) and a surfactant (polyglycerol ester of fatty acid, PGE 080/D). The detailed description of synthetic and purification procedures have been described by the authors of [61]. The chemical compositions of the three matrices are reported in Table 1. As it can be seen, they varied with respect to the relative amount of GMA and DVB.

In order to increase the water wetting properties of the matrices, the epoxy groups of GMA were hydrolyzed by refluxing the matrices in a solution of H_2_SO_4_ (0.1 M) in dioxane for 4 h. The porous matrices were then Soxhlet-extracted with water, then with THF and finally vacuum dried.

Samples were labelled with the simple code G*x*, where G stands for glycidylmethacrylate and *x* is the volume % with respect to total monomer and cross-linker volume. Consequently, the % volume of theDVB was the complement of the GMA.

#### 4.2.3. Dielectric Spectroscopic Measurements

Dielectric measurements were taken at frequencies between 1 kHz and 2 GHz using two Impedance Analyzers Hewlett-Packard mod. 4291A and mod. 4294A at the temperature of 25 °C, following the procedure described in detail elsewhere. Briefly, the samples, shaped as cylinders of 7 mm in diameter and 10 mm in height, were gently fitted in a measuring cell consisting in a short section of a cylindrical waveguide, directly connected to the Meter through an APC7 precision connector. The analyzer, in the frequency range investigated, measured the complex reflection coefficient Γ(ω), from which the complex dielectric constant $\varepsilon^{*}(\omega )$ was obtained using a calibration procedure performed with air, short connection and an aqueous electrolyte solution of known permittivity and electrical conductivity.

#### 4.2.4. FE SEM Measurements

Scanning electron microscopy (SEM) images were obtained with a LEO 1450VP operating at 10 or 20 kV. Field emission scanning electron microscopy (FE–SEM) images were obtained using a HR FESEM AURIGA (Zeiss, Jena, Germany). Prior to observation, fractured samples were mounted on aluminum stubs using carbon paste and sputter coated with a chromium layer using a turbo-sputter Q150T ES (Quorumtech, Lewes, UK). Micrographs at high magnification (×10^5^) of the microstructure of the matrices walls were taken in the InLens modality.

#### 4.2.5. DW-NMR Imaging Measurements and Processing

All measurements were performed on a Bruker 9.4 T Avance system, operated with a microimaging probe (10 mm internal diameter bore) and equipped with a gradient unit characterized by a maximum gradient strength of 1200 mT/m and a rise time of 100 μs. An imaging version of the pulse gradient stimulated echo (PGSTE) sequence with an echotime/repetition time *TE*/*TR*= 15/(5000−Δ)ms; diffusion time t = Δ in the range of 20–250 ms; gradient pulse duration δ = 2 ms; gradient strength g = 0 and 74 mT/m with gradient direction along the x, y and z axis; image matrix 128 × 128; field of view FOV = 0.8 cm; slice thickness STH = 1 mm; and number of averaged scans *NSA* = 32 was used to obtain the conventional mean diffusivity maps (*MD* maps) and *M*α maps, as previously described [27,29,62]. The temperature of each sample was fixed at 291 °K. All fitting procedures were performed by means of the Levenberg-Marquardt algorithm using homemade scripts in MATLAB.

## Figures and Tables

**Table 1:** Monomer and cross-linker relative content in the starting emulsion. Surface areas and average pore diameters <D_p_>, of the three matrices investigated.

Sample	GMA ^a^ (%vol)	DVB ^b^ (%vol)	PGE ^c^ (%vol)	Specific Surface Area m^2^/g	<D_p_> (m^2^/g) (A°)
G40	40	60	5	188	64
G60	60	40	5	100	107
G80	80	20	7	16	274

^a^ glycidylmethacrylate; ^b^ divinylbenzene (80 vol% m-(55 vol%) and p-divinylbenzene (25 vol%), the remainder being m- and p-ethylstyrene; ^c^ concentration expressed as weight % with respect to the total volume of organic phase.

## Data Availability

The datasets generated and analyzed for this study can be shared upon reasonable request to the corresponding author.
